# Factors that create trust in the donation after circulatory death process among surgical personnel: a qualitative descriptive study

**DOI:** 10.1186/s12913-026-14525-y

**Published:** 2026-04-14

**Authors:** Eva Lagging, Kjerstin Larsson, Ulla Forinder, Annika Tibell, Linda Gyllström Krekula

**Affiliations:** 1 Center for Health Care Ethics, LIME, Karolinska Institutet, Stockholm, 171 77 Sweden; 2 Perioperative Medicine and Intensive Care, Regional Donation Center Stockholm-Gotland, Karolinska University Hospital, Solna, Stockholm, 171 76 Sweden; 3 Department of Public Health and Caring Sciences, Public Health, Working Life and Rehabilitation, Uppsala University, Uppsala, 752 37 Sweden; 4 Department of Social Work, Criminology and Public Health Sciences, University of Gävle, Gävle, Sweden

**Keywords:** Donation after circulatory death (DCD), Organ procurement, Surgical personnel, Trust

## Abstract

**Background:**

The global scarcity of organs donated for transplantation has rekindled the interest in donation after circulatory death to augment the organ donation pool. However, there is a notable gap in our understanding of the attitudes and experiences of the surgical personnel involved in donation after circulatory death retrieval operations, which can adversely impact the success of donation after circulatory death programs.

**Methods:**

We conducted focus group interviews using open-ended questions both before and after surgical personnel participated in the donation after circulatory death pilot. Thematic analysis was applied to the focus group data.

**Results:**

Focus group responses generated one main theme: Trust in the donation after circulatory death process and four subthemes: Documents and education about donation after circulatory death; Intensive care unit handover to operation; Factors during the surgical process that strengthens team building; and Treating the donors and their families with dignity and respect.

**Conclusion:**

Trust in the donation after circulatory death process, which fosters a supportive work environment among surgical personnel, hinges on the implementation of national donation after circulatory death guidelines based on evidence, a comprehensive intensive care unit handover that elucidates knowledge about patient care details, a pre-surgery planning meeting that generates team spirit. Furthermore, it is perceived vital to treat the donor with the utmost respect and dignity, as would any other patient.

**Supplementary Information:**

The online version contains supplementary material available at 10.1186/s12913-026-14525-y.

## Background

The worldwide shortage of donor organs for transplantation has led to a resurgence in the practice of donation after circulatory death (DCD), a supplementary form of organ donation post-mortem [1]. DCD is an effective way to increase organ donation and is being adopted by an increasing number of countries [2]. According to the Global Observatory on Donation and Transplantation (GOAT), in 2023, deceased donation was conducted in 77 countries, with 75 performing donations after brain death (DBD) and 26 engaging in DCD [3]. In 2009, a third of the 27 member nations in the Council of Europe had active DCD programs with many others, including Sweden, planning to initiate such programs [4]. The attitudes and knowledge of health care professionals (HCP) involved in organ donations, influence organ donation rates [5]. Yet little is known about these factors among HCPs involved in DCD, particularly among surgery nurses and assistant surgery nurses.

## Methods

### Aim

This study aimed to investigate surgical personnel’s attitudes and experiences regarding the donation process, whether these attitudes and experiences altered after participation in a DCD pilot test, and to identify elements that contribute to trust in the DCD process.

### Study design

This was a focus group (FG) study with a qualitative descriptive approach. Focus groups are a recognized method for eliciting detailed discussion on specific topics [6]. The study did not assess causality; rather, it described attitudes before and after the pilot test of DCD. The reporting followed the Consolidated Criteria for Reporting Qualitative Research (COREQ), a widely accepted checklist for qualitative studies [7].

### Setting

**Fig. 1:**
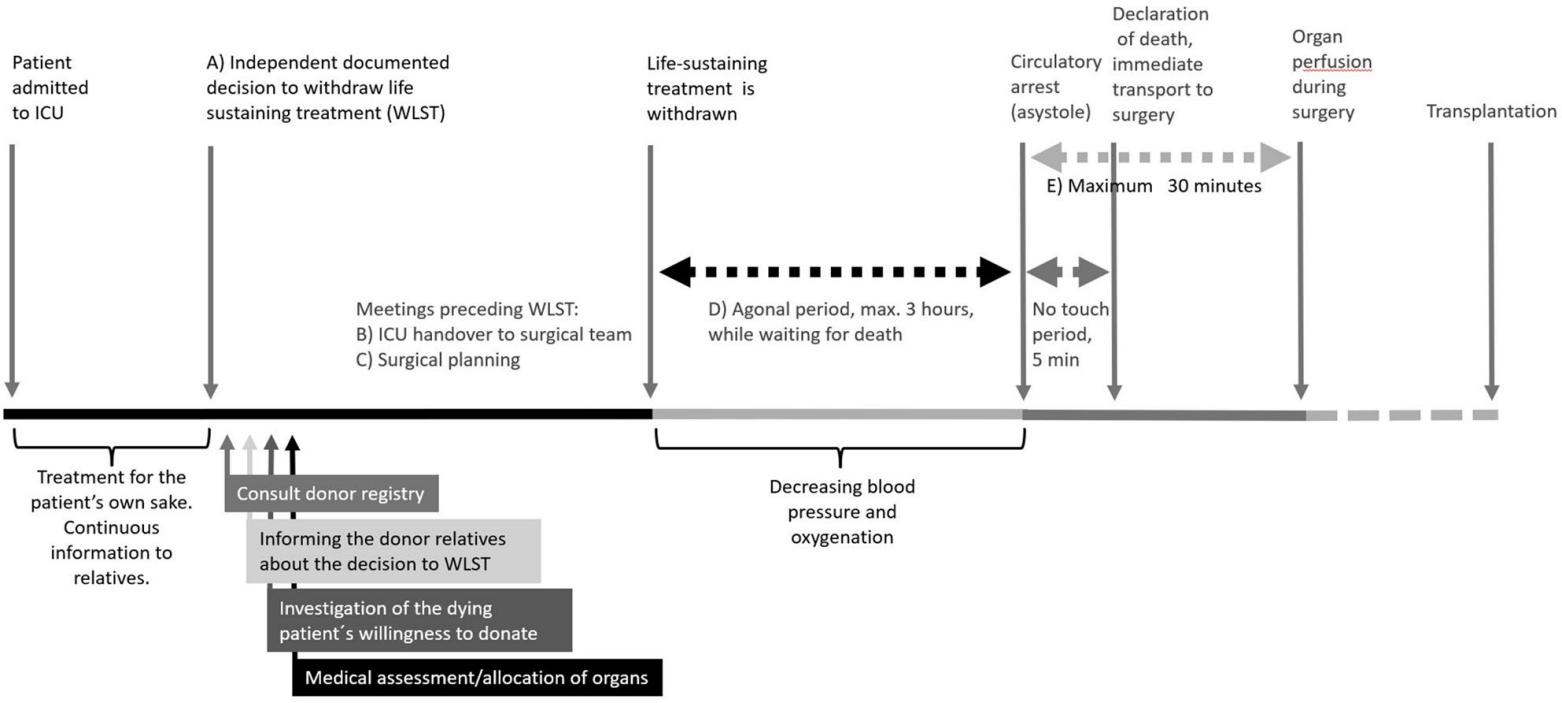
Flowchart of the controlled donation after circulatory death process

Following a series of state-of-the-art meetings with national and international experts, the National Council for Organs, Tissues, Cells and Blood of the Swedish Association of Local Authorities and Regions (SALAR), launched a project to explore the feasibility of implementing controlled DCD (category III) in Sweden. A multidisciplinary project group gathered knowledge and experience via international experts and site visits, developed a national guideline, trained local DCD teams, and initiated a DCD pilot test across six hospitals (Fig. 1). All four transplantation centers in Sweden took part in the pilot test. The pilot test period lasted for 12 months at which point the goal of the study, ten complete DCD processes, had been reached. During the pilot test, only kidneys and lungs were retrieved and transplanted.

### Participants

The FG interviews were conducted both before and after the pilot test, with homogenous sampling appropriate for focus groups [6], in this case HCPs involved in organ procurement surgery.

Thus, the FGs consisted of operating room nurses, assistant nurses, as well as some of the involved transplant surgeons and transplant coordinators from Sweden’s transplant centers.

Given that this was a clinical project aimed at introducing a new method, participation required nurses and assistant nurses with extensive DBD experience and sufficient motivation. Selection was performed by the DCD pilot hospitals, which assigned staff to the clinical teams during the pilot test.

The four FGs from before the pilot test were conducted when the teams attended a two-day DCD workshop and consisted of 6–13 participants (Table 1). The workshop began with all participants attending a course covering various aspects of DCD, providing foundational knowledge prior to the pilot test. The four FGs after the pilot test were performed when the participants attended a follow-up meeting and consisted of 4–9 participants (Table 1).

**Table 1:** Participant characteristics in focus groups

Total participants in focus groups	Before DCD pilot	After DCD pilot
	40*	29
**Gender**		
Female/Male	34/6	25/4
**Age**		
20–30	3	
31–40	11	
41–50	13	
51–60	11	
61-	1	
**Profession**		
Transplantation surgeon (S)	6	1
Transplantation coordinator (C)	11	13
Operation nurse (N)	14	10
Operation assistant nurse (AN)	9	5
**Years worked in profession**		
0–5	5	
6–10	7	
11–20	8	
21–30	12	
31–40	7	
**Years in current field**		
0–5	15	
6–10	7	
11–20	10	
21–30	3	
31–40	4	
**How much experience do you have working with DBD donation**		
Very large	14	
Large	5	
Relatively large	9	
Relatively small	6	
Small	1	
Very small	4	
**Have you decided if you want to donate your organs after death?**		
Yes, I have decided to donate	36	
Yes, I have decided not to donate	0	
No, I have not decided whether to donate or not	0	
I do not want to/cannot answer	1	
**How do you feel about working with DCD during the pilot test period?**		
Highly motivated and looking forward to working with DCD	35	
Basically, positive about working with DCD but still feel some hesitation	1	
Feel a relatively large resistance to working with DCD	0	
Feel great resistance to working with DCD	0	
Feel discomfort and/or dislike about working with DCD	0	
**Why are you part of your local DCD team?**		
I volunteered to be part of the team	17~	
I was asked to join the team and accepted	16~	
I was assigned a place in the team without having a choice of my own	0	

*One person never handed in the questionnaire but attended the focus group, ~ One participant gave two answers

### Data collection

A semi-structured interview guide with open-ended questions was used to ensure consistent coverage of topics across all groups. The interview guide was based on a patient case and included one part about the donation process at the intensive care unit (ICU): the decision to withdraw life-sustaining treatment (WLST), investigation of consent to organ donation, withdrawal of life-sustaining treatment, and the death of the patient. Another part dealt with the donor operation: HCPs reflections regarding the patients’ death, planning the operation, thoughts on how to achieve a good working environment, flow during the operation, and thoughts regarding organ function in the recipient (see supplementary information).

All participants taking part in the FGs before the DCD pilot test also answered a short questionnaire that included individual participant characteristics and their view on DCD ahead of working with it (summarized in Table 1). This data serves to describe the characteristics of the participants.

Focus groups were chosen as a central part of the study design as they facilitate collective discussion in which participants could critically examine and reflect on their viewpoints. This selection was integral to the study design. All focus groups were moderated by LGK and observed by UF, with interviews recorded and transcribed verbatim resulting in 600 pages, with a mean interview time of 132 min (min. 129 – max. 167). To ensure that a particular response cannot be linked to a specific participant, all study material was pseudonymized.

### Analysis

Thematic analysis was conducted using an inductive approach, with themes derived directly from participants’ accounts and grounded in the data [8]. The interviews from before and after the pilot test were analyzed separately in the following steps. The interviews were read and reread by EL and KL to capture the essence of the text. Texts that captured key thoughts or concepts of the study aim, were identified, and labeled with codes that described their content. The codes were then compared for similarities and differences and grouped into a structure of subtheme. All codes were utilized. The subthemes that emerged at the beginning of the analysis gradually evolved as new data were introduced. This iterative process was conducted by EL and KL until a shared understanding of the subtheme and theme structure was achieved. During the analysis process, the authors involved constantly moved back and forth between the parts - such as the codes - to the entire text to ensure coherence. The preliminary classification was then discussed among the research group until an agreement about the relevance of the structure was reached. In a final step, citations were selected illustrating the subthemes. After performing the separate analyzes the data from before and after the pilot test were compared to detect any changes in attitudes and experiences. Neither computer software was used for analysis and data storage, nor member checking was performed. Pilot tasting was not conducted. Field notes were not taken as the FG were audio recorded. The quantitative data from the questionnaire was analyzed and reported using descriptive statistics.

### Research team

The authors, all female, had diverse preconceptions of the studied phenomena. The lead author (EL) is a PhD student with experience of interviewing patient associations to draw on their members’ experience and has conducted individual interviews in an earlier study. Authors KL, LGK and UF are medical social workers and qualitative researchers, KL in chronic diseases and both LGK and UF in deceased organ donations. Author AT has long experience as a transplant surgeon, with a special focus on organ donation and ethics. Authors KL, LGK, UF and AT are all PhDs. LGK has worked in the field of organ donation since 2003 and is well known among professionals in Sweden. Shortly before the after-pilot test focus groups were conducted, she was appointed Head of a Regional Donation Centre, thereby assuming a managerial role over some study participants. For this reason, UF was present as an observer to minimize the risk of bias.

### Contact with the participants

Only LGK and UF had contact with the participants and UF only in connection with the study. LGK works with organ donation and thus to and from has contact with the participants.

### Ethical considerations

The interview questions may be perceived by the participants as personal and could thus constitute an infringement of their integrity. All participants were informed, both in written form and orally, that participation was voluntary, and that they had the right to withdraw at any time without explanation. Participants were also informed that the study material would be pseudonymized and remain strictly confidential throughout the study process. Thus, a particular response cannot be linked to a specific participant. Prior to the FG interviews, written consent was obtained from the participants. For those who participated in both the FG interviews before and after the DCD pilot test, one consent covered both FG occasions. Participants were offered psychological support post-interview if needed. None desired this. The study was approved by the Ethics Review Committee in Stockholm (D.nr. 2017/1704-31/2). This study does not involve organs/tissues procured from prisoners.

## Results

The thematic analysis resulted in one main theme: Trust in the DCD process, and four subthemes: (1) Documents and education about DCD; (2) ICU handover to operation; (3) Factors during the surgical process that strengthen team building; and (4) Treating the donors and their families with dignity and respect (Table 2).

**Table 2:** Relationship between theme and subthemes

Theme	Subthemes
Trust in the DCD process	Documents and education about DCD
	ICU handover to operation
	Factors during the surgical process that strengthen team building
	Treating the donors and families with dignity and respect

### Main theme – trust in the DCD process

The main theme in this study - Trust in the DCD process - represents the central meaning which has emerged from the data. The importance of trust summarizes the core aspects of the participants’ experiences and perspectives of DCD, which are reflected in every subtheme (se next paragraph). The main theme entails the trust that SP has in the professionalism of the ICU staff, and in the guidelines covering the whole donation process, from the patient being admitted to the ICU till the donor operation. It furthermore involves the trust that emerged through having the prerequisites of ensuring optimal preparation for the donor operation and fostering effective teamwork throughout the surgical process. A key factor in creating trust in the DCD process is the display of mutual respect among all parties – from patients and their families to ICU staff, and operating staff.

### Subthemes

The four sub-themes that emerged from the data are more specific and detailed expressions of distinct parts of the main theme. They show how the main theme, trust, is manifested in various situations, contexts, in relation to DCD.

### Documents and education about DCD

Before the pilot test, there was a demand for more detailed surgical documentation, which surgeons found impractical due to variations in surgical methods. After the pilot test, participants recognized that preparatory meetings with the entire surgical team adequately addressed this requirement.

*I think it might be good to continue to keep the protocol fairly non-detailed in terms of the detailed surgical technique. (S1)*

Participants´ views on the importance of documents and education were the same before and after the pilot test. Participants stated that the DCD workshop and the simulation were essential, as they provided good knowledge and reassurance about the organ retrieval surgery and fosters trust in the entire process. The DCD guideline was recognized for its solid foundation, drawing from international research and expert symposiums. Its contribution to uniform DCD implementation and the development of local checklists to streamline workflow also enhance its reliability.

*No*,* I think the guideline was generally good. I experienced for my own part that it became a kind of… an overview of the process where you had to pick out what was relevant for your own part. (C1)*

FGs revealed the necessity of clarifying to all involved parties, in guidelines and education, that the decisions to WLST and to investigate organ donation willingness are two distinct processes, ensuring that the WLST is never undertaken to facilitate organ donation (Fig. 1:A). This standpoint was as strong both before and after the pilot test.

*It’s true that it shouldn’t be perceived as that you switch off just because you need the organs. (N1)*

### ICU handover to operation

After the pilot test, the SP realized the importance of the handover meeting between ICU and surgical personnel, covering why the patient´s ended up in the ICU, their ICU journey, and that there was a well-founded decision to donate. This was seen as helpful in enhancing knowledge and confidence in the parts of DCD process that oneself was not involved in (Fig. 1:B). Participants expressed a desire for similar meetings in DBD cases.

*I think close contact with those I work with at ICU and that team is important. You listen and understand in a different way. (N2)*

Some staff wanted patient background information, while others saw it as advantageous but non-essential as they were used to emergency operations where such information is often lacking. These opinions were found among participants both before and after the DCD pilot test.

*If I must choose*,* I want information*,* but I don’t think it would affect the way I work if I didn’t have the information. (N4)*

Handover meetings were perceived as positive and helpful to ensure that all staff were on the same track, which was reinforced after the pilot test. Transplant surgeons with previous experience of DCD abroad mentioned how important these meetings were to ensure that all staff felt involved in the process and thus felt more secure with DCD donation. The SP, already before the pilot test, had high confidence in the ICU staff´s professionalism and in their competence in making medical evaluations of patients. This confidence was confirmed after collaborating with the ICU staff in the handover meetings.

*My own experience from this when I’ve worked with DCD in other places abroad… then you always had a joint meeting with the operating staff before WLST and someone from ICU… came over and you sat down beforehand got a brief history about the patient*,* what had happened so everyone knew what was relevant and I think that helped quite a lot instead of you don’t know anything about the patient. (S2).*

Prior to the DCD project participants had trust in and knowledge of the fact that ICU doctors have extensive experience of declaring patients dead based on indirect criteria regardless of whether organ donation is relevant or not. After the pilot test there was no change in the participants’ confidence regarding the fact that the patient was dead on arrisval at surgery. This confidence was confirmed by participating in the handover meeting, where they met the ICU physicians responsible for declaring the patient dead.

*We have confidence in the doctor who made this declaration of death. (S3)*

In addition, regarding confidence in death, participants already before the pilot test anticipated that death would be clear in the DCD context. They envisioned patients arriving in the operating room without life-support machines, without a pulse, electrical activity, reflexes, or breathing. This absence of vital signs in the DCD context, as opposed to DBD, confirmed the participants’ notion that the patient had indeed passed away.

*Yes*,* but then you’ll see. You have no breathing*,* no pulse*,* they are relaxed in a different way than living people are*,* even if they are sleeping. (N3)*

*No*,* but I think it might be visually easier to understand that this person is dead when they don’t come with any life support machinery. (AN1)*

### Factors during the surgical process that strengthen team building

A new aspect that emerged after the pilot test was that the waiting period after WLST, when waiting for the patient to die, allowed team members to focus on their own role during the surgery and fostered a team spirit through dialogue amongst them (Fig. 1:D). For them, it was important with continuous updates from the ICU on the patient’s status. After the pilot test, appreciation was expressed that they could continuously follow the patient’s status in real time through monitoring devices. However, the anticipation of a patient´s death felt strange to await. The extent of the ambivalence participants felt—between gearing up and motivating themselves for an important procedure, while at the same time empathizing with the relatives sitting at the deathbed, was something they did not anticipate before the pilot test.

*It feels very strange to stand and wait for someone to die somehow. I think it felt a bit strange at the time actually. Even if you have the whole picture completely clear to you of course. (N5)*

In the setting of DCD there is a limited time of 30 min for washing and dressing the donor, and for achieving cold ischemia. Prior to the pilot test, the participants were concerned that this time limit was too short. However, this concern diminished after the pilot test as participants found the allocated time to be sufficient (Fig. 1:E). They then noted that because everything was set up before the patient arrived, it went faster but calmer compared to an emergency operation.

*You don’t always know when an emergency comes in and when they are… in such a hurry. Here we still have a plan and have the right stuff up*,* hopefully. (N5)*

Because time is limited in DCD surgery, participants already before the pilot test recognized that a thorough preoperative planning meeting prior to WLST was essential. Such preparation helped ensure they were ready before the surgery began.(Fig. 1:C). It was important that the surgeon informed them of what instruments they needed during the surgery, so that these were displayed before the patient arrived in the operating room. This enabled them to reduce their pre-surgery stress and provided a sense of security.

*Very important*,* yes. Really. It was very reassuring to have this pre-meeting where everyone was involved and went through every step*,* and everyone felt safe and knew what they were going to do… (N6)*.

The pre-planning meeting also reinforced team dynamics where each member knew what was expected of them. That operating staff with no or little previous experience of organ donation operations now had the possibility to talk to surgeons and other experienced staff provided reassurance. The participants also wanted similar pre-operation planning meetings at DBD.

Based on previous experiences from other surgical procedures, the participants put forward the importance of the surgeon’s leadership during surgery. Already before the pilot test they realized that, given the time limitation in DCD, the utmost importance lay in the surgeon communicating clearly about what they are doing, intended to do and what they need. After the pilot test it was confirmed that the surgeon’s communication skills were crucial and facilitated the operation and created a positive atmosphere in the room.

*She (surgeon) tells you exactly what she is doing all the time. So*,* everyone in the operating room*,* the assistant nurse and everyone knows exactly where she is in the process.…but still so that everyone in the room can understand. (C2)*

A post-operative debriefing with the surgeon was requested, ideally occurring during wound suturing due to concerns about the possibility of regrouping the staff after surgery completion.

### Treating the donors and families with dignity and respect

The concepts of dignity, respect, and calmness consistently resonated with the participants both before and after the pilot test, highlighting the importance of these values throughout the DCD process. They underlined that donors must always be treated with the utmost respect, just like any other patient.

Furthermore, the SP brought up how important it was that relatives were given the opportunity to understand and accept how the patient’s status deteriorated and that finally there was nothing more to do. The relatives must also be informed that the dignity and respect that had been given to the patient at the ICU continues during surgery. It was essential to reassure relatives that the donor was properly cared for after the operation. This was done by informing them that the patient was washed, dressed in clean clothes, and that the surgical wound was bandaged, as after any operation.

*It is still someone who should be treated with respect. (N7)*

The operating personnel saw their role in the procurement process as a way to help donors fulfill their wish to donate their organs and help other patients to continue living. For the surgical nurses and assistant nurses, it was also considered valuable to receive information about how the organs function in the recipients.

*We must do everything to make it happen* [donation] *and do our best*,* so that the wishes of the donor or relatives are met. (C3)*

## Discussion

This study describes surgical personnel’s attitudes and experiences before and after the pilot test and identified factors that contribute to trust in the DCD process. Although causal inference is not possible, the findings provide insight into how participants described their attitudes before and after the pilot test. The results reflect the participants’ perspectives developed during the pilot year, and how these experiences influenced their views and approaches to DCD.

The study findings should be understood in the context of a clinically driven implementation project that was simultaneously subject to research. From a clinical perspective, involving motivated staff with experience in DBD may facilitate the introduction of a new method. However, this approach also implies a degree of selection bias, as participation in the pilot test requires a certain level of motivation. The high level of trust expressed after the pilot test should therefore be interpreted with caution. Nevertheless, it is noteworthy that participants frequently referred to their concrete experiences during the pilot test, and it is reasonable to assume that, had the process been perceived as inappropriate, trust levels would likely have been lower.

### Trust in the DCD process

Trust is a fundamental prerequisite for implementation of Donation after Circulatory Death (DCD). In our study, we found four subthemes that were important for the surgical staff to trust the DCD-process: - education grounded in well-found guidelines, - the handover from ICU to the surgical staff, - team building before and during surgery, and - treating donors and their families with respect. Our data also emphasizes the importance of the surgical pre-planning meeting and the meeting for ICU-to Operation handover. Interestingly, participants expressed a desire for similarly structured pre-operation planning meetings and ICU-to-Operation handover meetings also in donation after brain death (DBD) cases.

During the Swedish pilot test, only kidneys and lungs were retrieved. Recent literature highlights that trust in DCD may be challenged by the introduction of novel technologies such as normothermic regional perfusion. While these improve organ quality and allow retrieval of more organs, they raise ethical concerns that must be addressed transparently to maintain public and professional trust [4, 9–12].

Some individuals oppose organ donation due to ethical, moral, or religious beliefs, and for these individuals—both in the general population and among staff—greater knowledge does not reduce their resistance, as their objections are rooted in conscientious convictions [13]. These objections are based on differences in values and not on a lack of knowledge [13]. While fostering trust may be regarded as an important goal, it cannot be assumed among all healthcare professionals and must therefore be approached with humility and sensitivity [14]. In our study, trust emerges as a key outcome, as it underpins confidence that the donation process has been conducted appropriately. At the organizational level, it may bring tension between expectations that healthcare professionals fulfil their professional obligations and the need to recognize and accommodate moral discomfort as in opt-in models and conscience-based exclusions. Consequently, organizing care in ways that enable staff to articulate concerns and, where feasible, to refrain from participation when alternatives are available, may represent an important strategy for maintaining trust and supporting ethically sustainable practice [15]. However, in practice, opt-in models and conscience-based exclusions may be difficult to implement in organ retrieval surgery [16]. This is particularly the case during evening or night shifts, when staffing is often limited to one or two surgical teams responsible for managing emergency procedures.

### Knowledge, education and training promote trust

Lack of knowledge about DCD among involved staff may negatively affect donation rates [17, 18]. Prior studies demonstrate that education, comprehensive guidelines, and locally adapted checklists are important factors for successful implementation [1, 19–21]. The six hospitals selected for the DCD pilot tests were those with ICUs having enough patients suitable for controlled DCD and a culture embracing organ donation. Physicians, nurses and assistant nurses were chosen by the pilot hospitals to join the clinical teams during DCD testing. All participants attended the course organized by the national DCD project covering various aspects of DCD, providing foundational knowledge prior to the pilot test.

The participants emphasized that the DCD workshop and simulations organized before starting the DCD-pilot test were essential, providing valuable knowledge and reassurance about the organ retrieval surgery and fostering trust in the entire process. The DCD guideline was recognized for its solid foundation, drawing on international research and expert symposiums. It contributed significantly to achieving a uniform DCD implementation [22]. These guidelines also formed the basis for local checklists that helped streamline workflows and enhance reliability.

### Two separate processes and decisions

Among our participants, several aspects of trust were already present before the pilot test. Still, our participants stressed the importance of clearly communicating to all parties involved that the decision to withdraw life-sustaining treatment (WLST) and the investigation of organ donation willingness are two separate processes. The importance of ensuring that WLST is never undertaken to facilitate organ donation is a major ethical principle and, as underlined by many, any perceived conflict of interest can totally erode trust in the DCD process [1, 2, 17, 19].

Although the ethical separation between WLST and organ donation is well established in policies, our findings reinforce how surgical staff themselves emphasize and internalize this boundary as a condition for trust. This aligns with recent publications emphasizing that maintaining this separation is essential for preserving the legitimacy of the DCD process in the eyes of both professionals and the public [1, 2, 23–25]. This bottom-up ethical affirmation from surgical teams adds a valuable perspective beyond traditional top-down ethical frameworks.

Operating teams expressed strong confidence in the ICU staff’s professionalism and competence to determine when further intensive care treatment was futile and to declare death. This trust is particularly central in DCD, where death is determined based on circulatory criteria. However, others have reported that staff felt concern as the criteria for death used in DCD were perceived as vaguer than those in DBD [26]. In some countries WLST is performed in the operating room which may expose the surgical staff to an unusual and unfamiliar situation making them feel uncomfortable [2, 15]. Swedish guidelines instead recommend withdrawal at the ICU, an environment where WLST is common and staff is used to the procedure.

### ICU handover meetings as a tool for cross-disciplinary trust

An important shift after the pilot year, concerned the handover from ICU to the operating room. Surgical staff found these meetings more valuable than expected, particularly for understanding parts of the process they were not directly involved in. This supports previous studies demonstrating that interprofessional communication improves understanding of each other’s roles, strengthens team cohesion, and enhances patient safety [20, 27, 28]. It also illustrates how shared training and communication can harmonize the needs and expectations of different professional groups.

While interprofessional communication is a known facilitator of trust, the explicit recognition of these meetings as a trust-building mechanism—especially in the context of DCD—adds a new layer to the literature. Most existing studies focus on ICU or transplant coordination, but not on how structured handovers influence surgical team confidence and cohesion.

### Emotional ambivalence during WLST

Participants described the waiting period after WLST as a new and unfamiliar experience—waiting for the patient to die in ICU while simultaneously preparing for a critically important operation, not least for the recipients. Our participants described this experience as emotionally complex and unfamiliar. This duality—balancing clinical readiness with emotional discomfort—has not been widely documented in the literature. While ethical and logistical challenges of DCD are well known, the emotional and psychological experience of surgical staff during this specific waiting period is a relatively new contribution.

The fact that staff appreciated real-time information/monitoring of the patient’s status during this phase adds a practical and emotional dimension that has not been emphasized in prior studies.

### Factors during the surgical process that strengthen team building and trust

Operating on a donor in DCD differs fundamentally from any other surgery – in no other context do the surgical team operate on a deceased individual whose body looks deceased (non-circulated). Even at DBD, the experience is not the same, as the donor then appears alive when arriving at the operation treated with a ventilator.

Like others, we found that the surgical pre-planning meetings held prior to the WLST were considered crucial, helping each team member to understand their role and prepare adequately before the surgery began [15, 27, 29–31]. It was especially important that the surgeon communicated which instruments would be needed during the procedure, ensuring they were ready and available before the patient arrived in the operating room.

We and others have found that these meetings reinforced team dynamics, reduced pre-surgery stress, and provided a sense of security. Operating staff with little or no prior experience in organ donation surgeries especially appreciated the opportunity to consult with surgeons and more experienced colleagues, [15, 22, 30, 32–34]. In our study, the surgeon’s role in leading these meetings and clearly communicating their intentions during surgery is described as key to efficiency and calmness in the operating room. This is consistent with research on surgical teamwork, where clear leadership and role clarity are essential for patient safety and team dynamics [35–37]. Additionally, recent findings from the US National Heart, Lung, and Blood Institute workshop highlight the importance of structured communication and ethical clarity in DCD protocols, especially when using advanced perfusion techniques [38].

In our study, the concerns about urgency felt before the pilot year were not realized. The DCD teams concluded that this surgery is not comparable to acute cesarean sections or aortic aneurysms, as they thought before the pilot test. The careful preparations beforehand prevent haste and contribute to dignity and calm during the operation. This indicates that such preparations should be sustained even as DCD becomes standard practice. One participant described what happened in the operating room as a “wordless dance”.

The expressed wish for post-operative debriefing with the surgeon to reflect and learn from each DCD case is a constructive and forward-looking suggestion that could influence future best practices. While common in high-stakes surgical environments, their specific application to DCD procedures—where emotional, ethical, and logistical complexities intersect—are not previously well documented.

### An important ethical dimension – respect for donors and families

All participants in our study emphasized the importance of treating donors and their families with dignity and respect throughout the entire process. This included ensuring that families understood and accepted their close one would not survive and trusted that the donor was treated with dignity - even after death [4, 15, 36, 39]. This respect is not only an ethical obligation but also a prerequisite for morally justifiable donation.

Organ procurement can be seen as a way to honor the donor´s wish to help fellow human beings in need of a transplant [20, 30, 36]. In line with others, we found that receiving feedback on how the organs functioned in recipients was perceived as highly meaningful [15, 26] especially for surgical nurses and assistant nurses. This can be seen as a form of ethical feedback that reinforces a sense of purpose and contributes to a care-ethical climate, characterized by empathy and responsibility.

In summary, we believe that, for a successful implementation of DCD, it is important that all categories of staff involved trust the DCD process. Our study identified several factors important to surgical staff, some to our knowledge, which was not previously well described in literature. The results from this focus group study have influenced the broader implementation of DCD in Sweden. We believe that our results may also well contribute to the development of guidelines for DCD implementation in other countries.

### Methodological consideration

Several measures were taken to strengthen the credibility of the study findings. As noted, a homogenous sampling was used to obtain all categories of operating staff that worked clinically with DCD during the pilot test. Furthermore, authentic citations were used to illustrate the content of the subthemes. The study´s dependability was enhanced by using the same interview guide that covered the whole DCD process with all FGs. Dependability was also validated by a transparent description of the steps in the research process. Confirmability was promoted by the first, third, fourth and fifth authors’ pre-understanding of organ donation contributing to both broadening and deepening the analysis of the data. The second author had only a limited experience of working with transplantation acquired when contributing with the analysis and writing of a previous study [40], thus strengthening the analysis by minimizing the impact of the other authors’ pre-understanding. Transferability was promoted by meticulously describing both the characteristics of the participants and the context of the study.

There are limitations to this study that should be considered. These include that not all participants were able to participate in FGs both before and after the pilot test, sometimes due to change of workplace or not having had time off from regular work. Four new participants took part only in FG after the pilot test, replacing those who left due to change of workplace The participants in the focus groups consisted of dedicated teams recruited by the various hospitals included and additional participants could not be recruited. Those participants still involved in the DCD process from FG 1 were all contacted prior to FG 2. The greatest attrition occurred among transplant surgeons, with only one participating in a focus group after the pilot test. However, their positive attitude towards DCD prior to implementation was one reason for its introduction. Additional insights might have emerged with full participation, but this was not feasible.

In addition, there were about six times more female than male participants which reflects the composition of the teams. We explicitly acknowledge that this study design does not permit causal inference. Nevertheless, it allows for a descriptive comparison of attitudes before and after pilot test.

## Conclusion

Our study highlights the necessity of providing surgical personnel with comprehensive knowledge of the entire DCD process and creating the conditions needed to foster team spirit and trust in operating room staff. These components, combined with always treating donors with respect and dignity, fortify the DCD program’s integrity.

## Supplementary Information

Below is the link to the electronic supplementary material.

Supplementary Material 1: Addition 1 is the English translation of the interview guide focus groups

## Data Availability

The data used and analyzed in this study is not publicly available because the interview transcripts contain personal and potentially identifying information. Data from participant interviews is available from the corresponding author upon reasonable request.

## Abbreviations

DBD: Donation after Brain Death
DCD: Donation after Circulatory Death
ICU: Intensive Care Unit
WLST: Withdrawal of Life Sustaining Treatment
SP: Surgical personnel
FG: Focus Group(s)
HCP: Healthcare professionals
COREQ: Consolidated criteria for reporting qualitative research
