# Supplementary material for: Factors that create trust in the donation after circulatory death process among surgical personnel: a qualitative descriptive study
Source: BMC Health Serv Res. 2026 Apr 14;26:535. doi: 10.1186/s12913-026-14525-y (PMC13088567; doi:10.1186/s12913-026-14525-y)
Supplement: Supplementary file 1 — Supplementary Material 1: Addition 1 is the English translation of the interview guide focus groups [file 12913_2026_14525_MOESM1_ESM.docx]

**Interview guide DCD focus groups**

**Before the focus group**

- Participants are asked to sign consent forms.
- Participants fill in the form with background questions.
- Moderator, participant and observer introduce themselves to each other and the roles are defined. The time frame and other conditions of participation will be reviewed by the moderator.

**Part 1, spontaneous reflections**

- First, what are your spontaneous thoughts and feelings about the introduction of DCD in Swedish healthcare?
- What do you think are the biggest advantages/strengths of DCD?
- What do you think are the biggest disadvantages/risks of DCD?
- How do you personally feel about working with DCD?

The following questions are only asked in the second round of focus groups, after the end of the pilot period:

If you summarize your spontaneous reflections:

- What in the DCD protocol was most helpful?
- What was missing from the DCD protocol?
- Was there something that was incorrect, or that otherwise complicated the donation process in the DCD protocol?

**Part 2, questions related to patient cases**

The following patient cases are discussed by the participants both in focus group round 1, before the pilot period, and in focus group round 2, after the end of the pilot period. Different emphasis will be placed on the different parts of the patient case depending on whether the staff in the focus group come from intensive care or surgery/transplantation. However, all participants will be allowed to discuss all parts of the case, but the moderator will adapt the way the questions are asked, depending on whether they are about a part of the donation process that the group participates in or not.

**Initial medical information about the patient**

DAY 6 IN THE ICU: Johan Johansson, 50 years old, fell ill with a heart attack and cardiac arrest 6 days ago. Johan fell ill at home and his wife immediately called for an ambulance. The wife, with the guidance of the alarm operator at SOS alarm, tried to perform layman's advanced cardiopulmonary resuscitation (CPR). The ambulance arrived after about 20 minutes and the ECG (electrocardiogram) showed asystole (cardiac arrest). Advanced CPR was performed for an additional 30 minutes before the heart rhythm returned. Johan regained spontaneous circulation after about 50 minutes. Johan was admitted to the hospital and underwent a balloon angioplasty for a narrowing (dense stenosis) of the heart's left coronary artery trunk. He was then transferred to the intensive care unit for continued intensive care.

Relatives consisting of his wife and two adult children were continuously informed of Johan's serious condition. After a few days, the doctors in charge emphasized that the prognosis was extremely poor as Johan had suffered severe extensive ischemic damage to the brain.

After a few days of care, Johan was deeply unconscious (RLS 8) without brainstem reflexes. Computed tomography (CT, X-ray examination) of the brain showed signs of severe damage consistent with extensive brain damage due to ischemia. SEP (sensory evoked potentials) showed a lack of N20 potential (sensory cortex) on both sides.

**Conversation about withdrawal of life sustaining treatment (breakpoint)**

*On day 6 in the intensive care unit, the physicians in charge for the patient make the decision to withdraw the life-sustaining treatment as it is no longer considered to benefit the patient medically and the neurological prognosis is very gloomy. In a breakpoint conversation* *with the wife and the two children, the family was informed about the basis for the decision to withdraw the life-sustaining treatment (ventilator, norepinephrine, etc.). Furthermore, the next of kin receive information about how the withdrawal actually takes place.*

**Questions to the participants:**

- Tell us, how do you think you would feel about entering this breakpoint conversation?
- How would you reason and prepare for the conversation?
- What do you think is important for how a breakpoint conversation is received by relatives?
- What do the relatives need in that situation?
- If you believe the patient can donate organs later in the process, does that change anything?
- How do you view the involvement of a transplant coordinator in this situation (and possibly a donation specialist nurse/DOSS)?
- What in the DCD protocol was helpful or complicated the breakpoint conversation? (To be asked at focus group round 2, after the end of the pilot period.)

**The participants are then given two descriptions of how the conversation with Johan's relatives went:**

**Then imagine that...**

1. The communication between the doctors responsible and the family has been clear and honest throughout the period of care. Relatives have continuously taken in the information, and they have a great acceptance and insight into the situation.

**Questions to the participants:**

- How does this affect your experience of the situation?
- How does this affect the way you prepare for the conversation?

**Then imagine instead that...**

1. Relatives have questioned whether the best care that can be given to Johan has really been given to him. The family strongly questions the doctors' decision to withdraw treatment.

**Questions to the participants:**

- How does this affect your experience of the situation?
- How does this affect the way you prepare for the conversation?

**Investigation of the willingness to donate (consent conversation)**

*In a new conversation – consent conversation* *– relatives are informed about the possibility of supplementing the withdrawal and end-of-life care, with Johan donating organs after his death. Relatives are told that the donor registry has been consulted and that Johan is not registered. The doctor explains what it means to become a donor.*

**Questions to the participants:**

- Tell us, how do you think you would feel about entering into such consent investigation?
- How would you reason and prepare for such a conversation?
- What do you think is important for how the conversation about donation (DCD) is received by relatives?
- Could it be of importance if it is the same doctor who decided to discontinue the life-sustaining treatment that is also having the conversation about donation?
- What in the DCD protocol was helpful or complicated the breakpoint conversation? (To be asked at focus group round 2, after the end of the pilot period.)

**The participants are then given two descriptions of how the conversation with Johan's relatives went:**

**Then imagine that...**

1. Relatives already know that Johan is willing to donate. The family is in favor of the possibility of donation after the withdrawal of life-sustaining treatment.

**Questions to the participants:**

- How does this affect your experience of the situation?

**Then imagine instead that...**

1. Relatives do not know Johan's wishes regarding donation after his death. The family also question why the treatment should be withdrawn and they are upset about how the issue of donation is raised in this situation at all.

**Questions to the participants:**

- How does this affect your experience of the situation?
- What should you think about, and do, in this situation?

**Planning for withdrawal of life-sustaining treatment**

*After consent for donation has been obtained, and a medical assessment has been carried out by the transplant unit that accepts Johan as donor and recipients have been identified,* *the withdrawal is planned for, but due to heavy workload on the withdrawal team, the discontinuation is postponed until late evening.*

**Questions to the participants:**

- What should you think about before the withdrawal?
- What is important from a personnel point of view?
- What is important from a family point of view?
- What in the DCD protocol was helpful and what was difficult, when planning for the withdrawal? (To be asked at focus group round 2, after the end of the pilot period.)

**The participants are then given two descriptions of the process:**

**Then imagine that...**

1. Relatives are satisfied with the decision to carry out donation and remain in the intensive care unit while waiting for the withdrawal. The family is calm and awaits additional relatives who are on their way.

**Questions to the participants:**

- How does this affect your experience of the situation?
- What should you think about, and do, in that situation?

**Then imagine instead that...**

1. Relatives have with hesitation accepted the decision to discontinue life-sustaining treatment and they have, after discussions, with obvious hesitation, interpreted Johan's willingness to donate positively. When it becomes clear that the withdrawal is postponed for many hours, the family hesitates to proceed with the donation. The family now wants closure immediately.

**Questions to the participants:**

- How does this affect your perception of the situation?
- What should you think about, and do, in that situation?

**Withdrawal of life-sustaining treatment**

*At 23.00, all life-sustaining treatment is withdrawn. The ventilator is turned off, the patient is extubated, and the norepinephrine infusion is turned off. The family will be present when the withdrawal is carried out.*

**Questions to the participants:**

- What would it be like for you to be present in this situation? What is your role/task in that situation?
- What is important for the different categories of staff who are present in the room or who are otherwise part of the donation process?
- What is important to the relatives?
- What in the DCD protocol was helpful or difficult during the interruption? (To be asked at focus group round 2, after the end of the pilot period.)

**The participants are then given two descriptions of the process:**

**Then imagine that...**

1. After the withdrawal, the situation is calm and relatives sit in the room and hold Johan's hand.

**Questions to the participants:**

- What would it be like for you to be present in this situation?
- Your reflections, thoughts?...
- How can you take care of the relatives in the best way?

**Then imagine instead that...**

1. Immediately after extubation, the patient has an unclear airway, a lot of mucus, a rushed and very labored breathing. The family becomes upset at this point and demands that "something be done".

**Questions to the participants:**

- What would it be like for you to be present in this situation?
- Your reflections, thoughts?...
- How can you take care of relatives in the best way?

**The patient dies**

*75 minutes after the withdrawal, the arterial pressure shows no measurable pressure. After 5 minutes of no-touch (when the patient is completely without medical interventions), the intensive care physician in charge states that the patient shows all signs of being dead* *(indirect criteria) and the patient is declared dead according to current procedures. The staff informs the family that Johan is dead and that he therefore needs to be taken immediately for surgery in order for the donation to be possible.*

**Questions to the participants:**

- What would it be like for you to be present in this situation?
- Would you trust that Johan was dead?
- How can you take care of relatives in the best way?

**The participants are then given two descriptions of the process:**

**Then imagine that...**

1. Relatives say a short goodbye to Johan and the staff quickly transports the patient to the operating theatre.

**Questions to the participants:**

- What would it be like for you to be present in this situation?
- Your reflections, thoughts?...

**Then imagine instead that...**

1. Relatives refuse to leave Johan. "What if he's not really dead?" the wife shouts.

**Questions to the participants:**

- What would it be like for you to be present in this situation?
- Your reflections, thoughts?...
- How can you take care of relatives in the best way?

**Donor operation**

*The deceased patient is rapidly transferred to the operating room where the surgical team is waiting. The patient is quickly moved to the operating table. The transplant surgeon in charge checks all documents including the death certificate. The operation needs to start immediately as it requires rapid surgery, to be compared to a catastrophic cesarean section or acute ruptured aortic aneurysm.*

**Questions to the participants:**

- What would it be like for you to be present in this situation?
- What should the transplant surgeon think about before the operation?
- What is important for the different categories of staff present?
- How can you handle the surgery so that it is perceived as respectful by everyone who attends?
- What is important for the relatives to know about the operation?

**The participants are then given two descriptions of the process:**

**Then imagine that...**

1. The surgical team quickly begins the operation and all staff in the room are well aware of how the procedure should be carried out. The surgical team works systematically and methodically to take care of the organs.

**Questions to the participants:**

- What would it be like for you to be present in this situation?
- Your reflections, thoughts?...

**Then imagine instead that...**

1. The assistant nurse in the operating room asks anxiously "do we know that the patient is really dead"? In addition, after a while, the operating room nurse questions the operator's "technique", which she perceives as "sloppy".

**Questions to the participants:**

- What would it be like for you to be present in this situation?
- Your reflections, thoughts?...

**Recipients of organs**

*After an independent decision was made to withdraw life-sustaining treatment, the transplant coordinator on duty was contacted. An initial medical investigation was initiated which showed no contraindications to DCD. Once consent for donation had been obtained, the investigation was intensified, and the donor was deemed medically fit and recipients were appointed.*

*The recipients of organs have previously been informed by letter that they may be offered a kidney or lungs from a donor who has died as a result of circulatory arrest. They have also been told that these organs work just as well as those from today's donors. However, for kidney recipients, it may take a little longer for the kidney to function fully, and that a limited period of dialysis may therefore be necessary. Furthermore, they have been told that regardless of DCD or DBD, the same strict quality criteria apply.*

*In the information letter, the recipients were encouraged to give feedback to the named contact person, if they* ***did not*** *wish to be transplanted with a DCD organ.*

**Questions to the participants:**

- How do you feel about everyone who is waiting for a kidney/lung being informed that they can get a DCD organ?
- What positive/negative consequences can you see from informing the recipients?
- Should recipients be offered the option of declining a DCD kidney/lung, during the pilot period - or always? If so – why/why not?
- If the recipients *are not* informed – what consequences could this have?
- What would it be like for you to call in a recipient for a transplant with DCD kidney/lungs?
- What would it be like for you to transplant a patient with DCD kidney/lungs?
- How confident are you that the kidneys/lungs are working well?
